# Reevaluating Golgi fragmentation and its implications in wound repair

**DOI:** 10.1186/s13619-024-00187-w

**Published:** 2024-02-13

**Authors:** Chandra Sugiarto Wijaya, Suhong Xu

**Affiliations:** 1 Department of Burns and Wound Repair and Center for Stem Cell and Regenerative Medicine, Second Affiliated Hospital, Zhejiang University School of Medicine, Hangzhou, 310058 China; 2 International Biomedicine-X Research Center of the Second Affiliated Hospital, Zhejiang University School of Medicine and the Zhejiang University-University of Edinburgh Institute, 718 East Haizhou Rd., Haining, Zhejiang 314400 China

**Keywords:** Golgi apparatus, Membrane repair, Trafficking, Wound repair and regeneration, Golgi fragmentation, Apoptosis, Cell division, Cancer, Neurodegenerative disease

## Abstract

The Golgi Apparatus (GA) is pivotal in vesicle sorting and protein modifications within cells. Traditionally, the GA has been described as a perinuclear organelle consisting of stacked cisternae forming a ribbon-like structure. Changes in the stacked structure or the canonical perinuclear localization of the GA have been referred to as “GA fragmentation”, a term widely employed in the literature to describe changes in GA morphology and distribution. However, the precise meaning and function of GA fragmentation remain intricate. This review aims to demystify this enigmatic phenomenon, dissecting the diverse morphological changes observed and their potential contributions to cellular wound repair and regeneration. Through a comprehensive analysis of current research, we hope to pave the way for future advancements in GA research and their important role in physiological and pathological conditions.

## Background

**Fig. 1:**
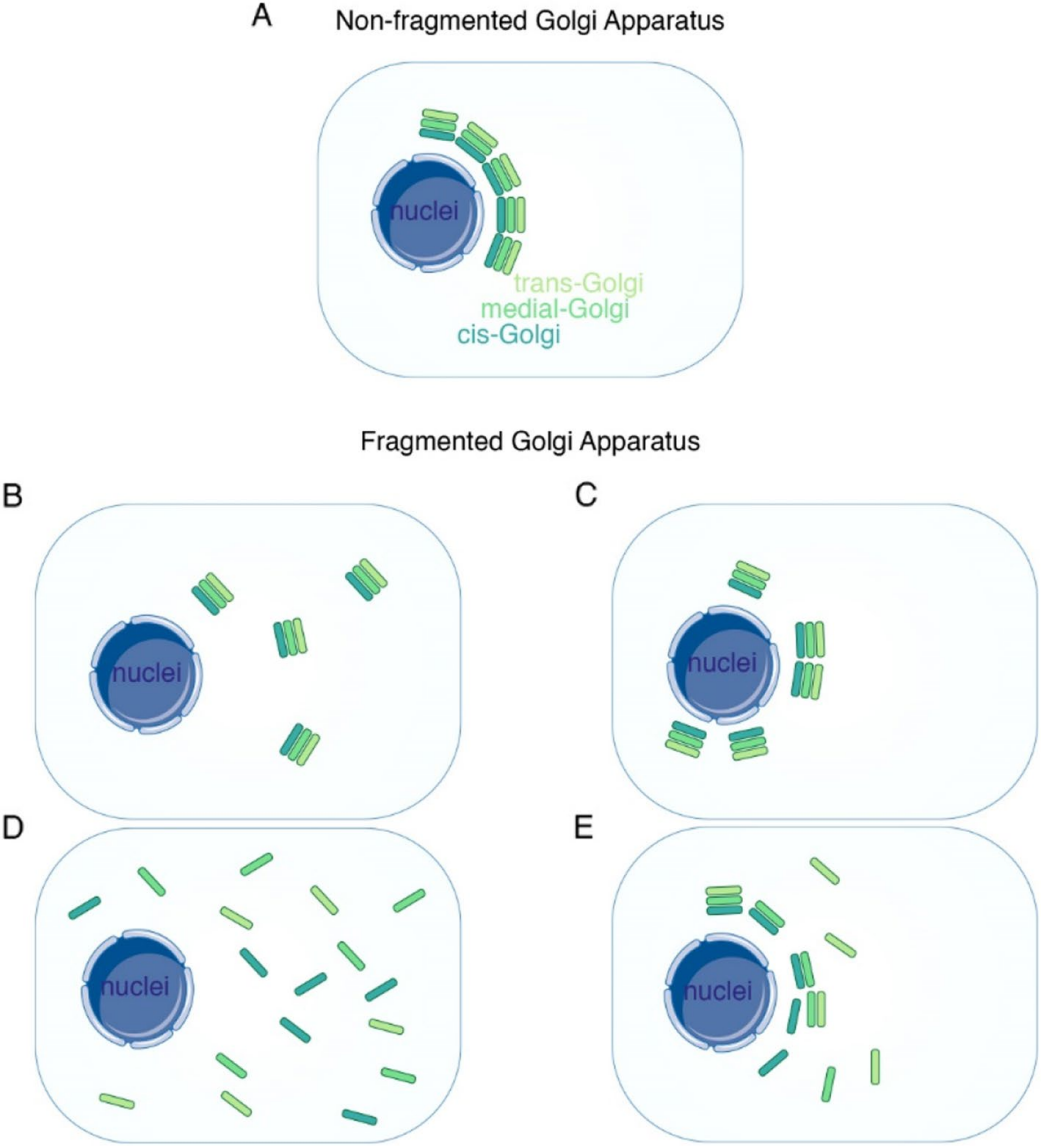
Illustration of the GA structure and distribution. **A** The canonical GA is localized in the perinuclear region and maintains a stacked and ribbon-like structure. **B**-**E** Fragmented GA exhibits various structures. **B** maintaining a stacked structure but losing the Golgi ribbon structure. **C** maintaining a stacked and ribbon-like structure but losing the polarity. **D** experiencing the loss of both the stacked cisternae and ribbon-like structure. **E** GA displays disorientation and decreased stack count

The Golgi apparatus (GA) is a cytoplasmic structure primarily located near the nucleus and is present in virtually all cell types (Bentivoglio 1999; Han et al. 2013). It comprises stacked cisternae, including the cis, medial, and trans compartments (Linstedt 1999; Lowe 2011; Nakamura et al. 2012). In mammalian cells, these GA stacks are interconnected laterally, forming a ribbon structure on the perinuclear region (Klumperman 2011) (Fig. 1A). The GA plays a crucial role in regulating intracellular transport processes. Newly synthesized proteins from the endoplasmic reticulum (ER) concentrate near the GA for sorting and undergo protein modifications, such as glycosylation, leading to the formation of secretory granules directed toward the plasma membrane (PM) (Jamieson and Palade 1967; Palade 1975).

**Fig. 2:**
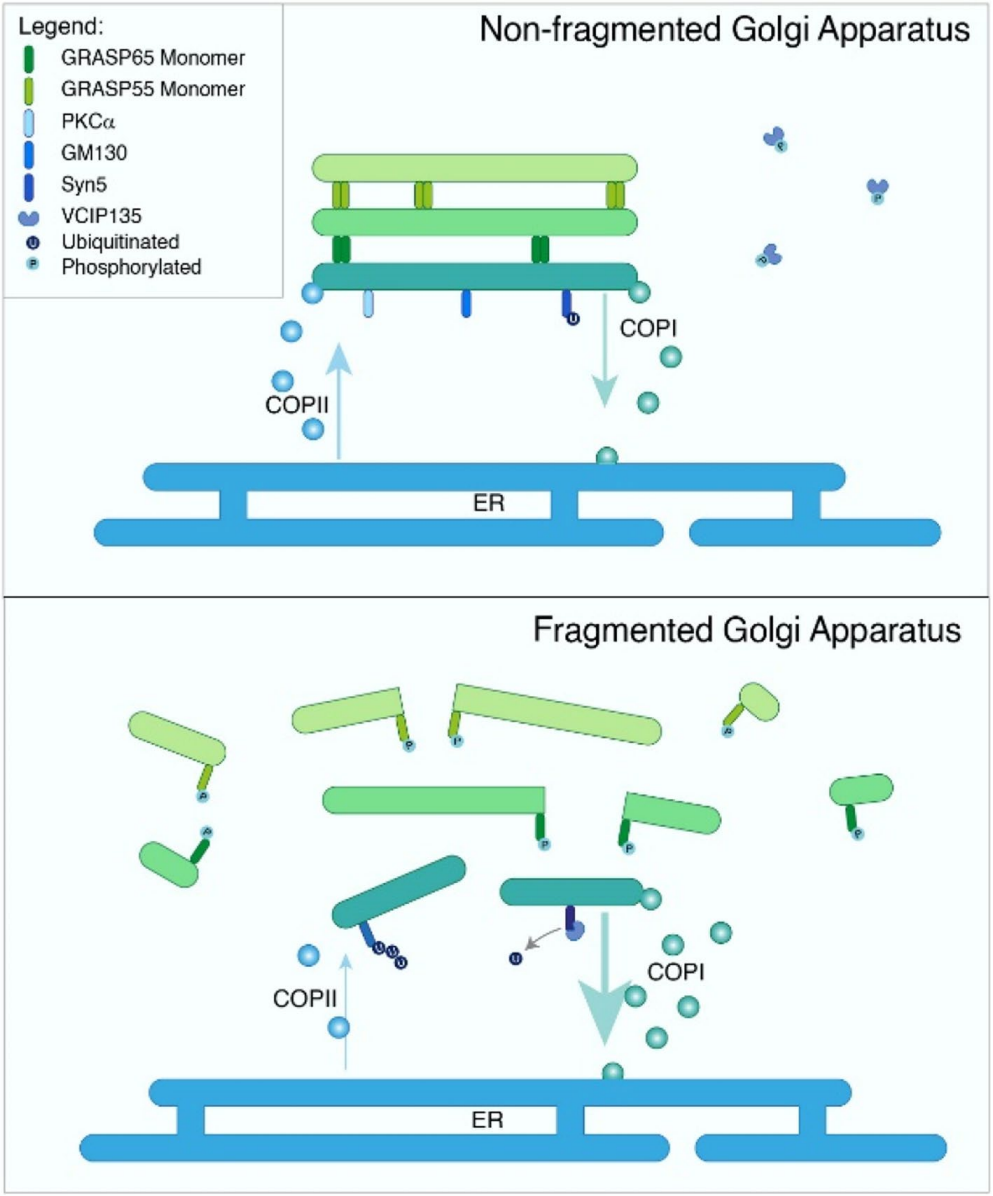
Illustrates the canonical mechanism for maintaining GA structure. Top, canonical GA maintains a stacked cisternae structure through the assistance of the structural proteins and regulates the balance of retrograde and anterograde transport to and from ER. In normal GA, GRASP 65 and GRASP 55 exist as dimers, providing adhesive force to maintain the stacks. Syntaxin5 (syn5) plays a crucial role in the assembly of vesicular-tubular pre-Golgi intermediates and cargo delivery to the Golgi. GM130, working alongside giantin and p115, acts as a tether, facilitating vesicle fusion to the GA. During GA fragmentation, GRASP 65 and 55 become phosphorylated, existing as monomers. GM130 undergoes polyubiquitination, targeting it for proteasomal degradation, leading to decreased vesicle fusion with the GA membrane. Syn5 is deubiquitinated by VCIP135, impairing its interaction with BET1 and promoting GA fragmentation. Below, GA fragmentation is an accumulative result that causes unbalanced retrograde and anterograde transport. A small arrow indicates decreased ER-Golgi anterograde transport. A large arrow indicates increased retrograde ER-Golgi retrograde transport. In canonical GA, the system should maintain the sorting, organized trafficking, and protein modification mechanism in the cell. Conversely, in the fragmented GA, there are defects in sorting, accelerated trafficking, and impaired protein modifications

The maintenance of efficient anterograde and retrograde transport between the ER and GA is essential for sustaining the structural integrity of the GA cisternae (Fig. 2). For instance, the inhibition of Arf1 GTPase activation has been shown to preserve the stacked GA structure during the mitotic phase (Altan-Bonnet et al. 2003, 2006; Xiang et al. 2007). Conversely, treatment with Brefeldin A (BFA), inhibiting COP II vesicle transport from the ER to GA by locking Arf1 in a GDP state (Chardin and McCormick 1999), disrupts the balance between anterograde and retrograde transport, leading to GA dispersal and retrieval to the ER (Sciaky et al. 1997). Besides the importance of ER-GA transport in maintaining GA integrity, GA resident proteins also contribute to maintaining cisternal adhesion, thereby ensuring the integrity of the GA structure (Tang et al. 2010). Consequently, the diverse distributions of GA in various cell types raise intriguing questions about the underlying mechanisms and their functional implications.

The non-perinuclear distribution of GA is commonly referred to as fragmented GA and has been observed in various organism fields (Jackson 2009; Wooding and Pelham 1998) (Table 1) (Fig. 1B-E). In the case of *Pichia pastoris (P. pastoris),* although the GA exhibits a fragmented appearance while still maintaining a stacked structure, it is neither ribbon-like nor localized to the perinuclear region (Mogelsvang et al. 2003; Suda and Nakano 2012) (Fig. 1B). In *Saccharomyces cerevisiae (S. cerevisiae)*, the GA does not form stacked cisternae as observed in mammalian cells. Instead, it exists as a non-perinuclear membrane structure, with the cis, medial, and trans cisternae evenly distributed in the cytosol (Fig. 1D). This is a distinct GA morphology and distribution in *P. pastoris* and *S. cerevisiae* are believed to have evolved from different ancestors, contributing to their unique characteristics (Papanikou and Glick 2009). Interestingly, adult *Caenorhabditis elegans* (*C. elegans*) epidermal cell (hyp7) also exhibits a non-perinuclear distribution of the GA (Zhu et al. 2016). Simultaneous observation of markers for the cis, medial, and trans cisternae in *C. elegans* reveals the presence of stacks structures (Meng et al. 2023).

**Table 1:** Comparison of GA morphology and role in different cell types

**Organism**	***S. cerevisae***	***P. pastoris***	***C. elegans***	***N. tabacum***	***H. Sapiens***				
**Cell examined**			Adult hyp7	BY-2	HT-29	SU.86.86	aRG	Myocytes	Myoblast
**Cell Features**	Mono-nuclear	Mono-nuclear	Multi-nucleated, Syncytium, Fully differentiated	Mono-nuclear	Mono-nuclear	Mono-nuclear	Mono-nuclear, stem cell	Multi-nucleated, fully differentiated	Mono-nuclear, stem cell
**GA distribution**	NP	NP	NP	NP+P	P	NP	NP+P	NP+P	P
**GA Features**	non-stacked, not divide during mitosis	stacked, not divide during mitosis, Generated from tER	stacked, matured	Stacked, preserved during mitosis.	Fragmented	Fragmented	Apical positioned	Fragmented	Stacked, ribbon like
**Role**	sorting station for both secretory and endocytic cargoes	Peroxisome function, Autophagosome formation	Promotes PM repair	Promotes cell wall and cell plate formation during cytokinesis	Promotes metastasis	Promotes metastasis	Controls bRG formation, Cell Polarity	Repair PM upon mechanical stress	Cell migration
**References**	Day et al. 2018; Suda and Nakano 2012	Manjithaya et al. 2010; Suda and Nakano 2012	Altun and Hall 2009; Meng et al. 2023; Zhu et al. 2016	Neumann et al. 2003; Rosquete et al. 2018	Bui et al. 2021; Kellokumpu et al. 2002; Petrosyan and Cheng 2013	Bui et al. 2021	Taverna et al. 2016; Xie et al. 2018	Quassollo et al. 2015; Ralston et al. 2001; Percival and Froehner 2007; Yadav et al. 2009	Percival and Froehner 2007; Yadav et al. 2009

*P* Perinuclear, *NP* Non-perinuclear, *NP* + *P* NP and P

Non-canonical GA distributions are also observed in mammalian muscle cells, neurons, and urothelial cells (Table 1). Skeletal muscle cells are multi-nucleated and display a non-perinuclear distribution of the GA. These non-perinuclear GA in neurons and skeletal muscle cells can be visualized using markers such as MANII and TGN38 (Quassollo et al. 2015). It is noteworthy that these non-perinuclear GA are generally smaller in size compared to the perinuclear GA. The term “Golgi outpost” (GOP) is commonly used to describe the non-perinuclear GA in both neuronal and muscle cells that are located far away from the nucleus (Valente and Colanzi 2015; Valenzuela et al. 2020). GOP is generated from the somatic GA through a sequential process involving tubulation, elongation, and fission (Quassollo et al. 2015). The fragmented GA in the urothelial cell can be observed in both normal and cancerous cells, where the structure of the fragmented GA varies with disease progression (Table 1). The GA structure and its regulation have recently been extensively reviewed elsewhere (Li et al. 2019; Lowe 2011; Makhoul et al. 2019; Martinez-Menarguez et al. 2019). This review focuses on the dynamic regulation of fragmented GA in various physiological and pathological conditions, emphasizing its role in regulating cellular wound repair and regeneration.

## GA fragmentation under normal physiological conditions

The GA undergoes a remarkable transformation during cell division, where its signature stacked cisternae unravel into ribbon-like structures, eventually forming scattered vesicles. This process, known as mitotic GA fragmentation or disassembly, is essential for ensuring the equal distribution of the GA to the daughter cells (Altan-Bonnet et al. 2006; Colanzi and Sütterlin 2013). Mitotic GA fragmentation involves the breakdown of cisternal stacks and the lateral connection, resulting in tubule-vesicular structures distributed evenly in the cytosol (Lucocq and Warren 1987; Sutterlin et al. 2002). Ribbon unlinking, a process regulated by Brefeldin A ADP-Ribosylation Substrate (BARS) and the phosphorylation of GA structural proteins GRASP55 and GRASP65, plays a crucial role in this phenomenon (Mascanzoni et al. 2022; Valente and Colanzi 2015).

Mitotic kinases, such as polo-like kinase and Cdk1, drive the phosphorylation of GRASP65, leading to the fragmentation of the GA (Sengupta and Linstedt 2010) (Fig. 2). GRASP55 phosphorylation, regulated by MAP kinase, also results in GA unstacking, forming structures termed “Golgi Blobs” (Colanzi and Sutterlin 2013). Importantly, the fragmentation of the GA during mitosis is reversible, initiating GA reassembly after cell division. NSF (N-ethylmaleimide-sensitive factor)-mediated membrane fusion re-forms the tubular-reticular elements of the GA, eventually restoring cisternae. The phosphorylation of VCIP135 by CDK1, reversed at the end of mitosis, activates p97/p47 and promotes GA reassembly (Valente and Colanzi 2015). Additionally, the dephosphorylation of GRASP55 by mTORC1 (Nuchel et al. 2021) and GRASP65 by PP2A contribute to the reassembly of GA stacks (Fig. 2).

GA fragmentation is traditionally associated with accelerated transport but impaired glycosylation (Haukedal et al. 2023). However, GA fragmentation induced by the depletion of GRASP55 and GRASP65 proteins resulted in accelerated transport while impairing the cell surface glycosylation (Ahat et al. 2022; Xiang et al. 2013). In Alzheimer’s disease (AD), Cdk5-induced GA fragmentation mediated by GRASP65 protein promotes amyloid precursor protein trafficking (Zhang and Wang 2020). In addition, under neuronal excitation, these fragmented GA contribute to the glycosylation of the newly synthesized proteins from the ER to promote secretion (Govind et al. 2021). GA fragmentation is observed in these protrusions, indicating GA fragmentation in the cell-cell communications (Kreft et al. 2022) (Table 1).

The GA undergoes significant changes in both structure and function under various stress conditions, such as DNA damage, energy and nutrient deprivation, abiotic stress, oxidative stress, aging, and pro-apoptotic conditions (Li et al. 2019). These alterations can occur through microtubule disorganization, altered protein phosphorylation, or degradation of key Golgi structural proteins. Additionally, the GA interacts with numerous signaling molecules, suggesting its potential role in sensing and transmitting stress signals within the cell (Alvarez-Miranda et al. 2015).

For example, in the process of apoptosis, the GA undergoes irreversible fragmentation, a phenomenon distinct from the reversible fragmentation observed during mitosis. Morphological changes in GA during apoptosis include swelling, loss of its ribbon-like structure, and the failure to maintain the perinuclear distribution (He et al. 2020). Caspase2 and Caspase3 are key players in GA fragmentation during apoptosis, inducing cleavage of resident proteins such as GM130, p115, and Golgin160 (Chiu et al. 2002). The cleavage results in the dispersal of the GA, contributing to the distinctive morphological changes observed during apoptosis. Importantly, GA fragmentation occurs during apoptosis before the cytoskeleton rearrangements (Mukherjee et al. 2007), setting it apart temporally from the regulatory mechanism of fragmented GA in cancer progression. Understanding the relationship between GA morphology and apoptosis could provide insights into why certain cell types retain a fragmented GA, though the beneficial role of this altered GA structure in preventing cell death remains poorly understood.

## GA fragmentation under pathological conditions

GA fragmentation has emerged as a distinctive feature in cancer progression and is recognized as a hallmark of the malignancy (Petrosyan 2015). Specific cancer cells, such as HT-29 colon tumor cells and SU.86.86 pancreatic cancer cells, exhibit a fragmented GA morphology (Bui et al. 2021; Kellokumpu et al. 2002) (Table 1). Under normal circumstances, GA undergoes fragmentation during the G2 phase of the cell cycle and restores its stacked perinuclear ribbon structure after mitosis. However, in malignancy, cancer cells maintain the fragmented GA morphology during the G2 phase, indicating an aberrant cellular state (Corda et al. 2012).

The motor protein NMIIA, localized in the cytosol and the GA, regulates vesicular trafficking within the cell (Togo and Steinhardt 2004). Inhibition of NMIIA using blebbistatin or siRNA has restored GA morphology in HT-29 colon tumor cells (Petrosyan and Cheng 2013) (Table 1), suggesting NMIIA-dependent regulation in GA fragmentation during cancer progression. Additionally, Petrosyan and Cheng showed that GA fragmentation induced by COP II inhibition (knockdown or BFA treatment) is also NMIIA-dependent (Fig. 2). While NMIIA emerges as a promising candidate for cancer therapy, further validation is required, as NMIIA is not exclusively localized to the GA, necessitating an exploration of potential side effects associated with its inhibition. Despite morphological evidence showing fragmented GA in multiple cell lines, the molecular mechanism underlying the relationship between GA fragmentation and cancer progression remains poorly understood.

Efforts have been invested in inducing GA fragmentation as a strategy to trigger cancer cell death. Inhibition of the Golgin protein GM130 impaired gastric cancer cell angiogenesis and metastasis capability (Zhao et al. 2015). GM130 degradation, mediated by the 26S proteasome, induced GA dispersal and cell death in myeloma cells, indicating a protective role for GM130 in maintaining GA structure (Eisenberg-Lerner et al. 2020). However, conflicting outcomes were reported, as a clinical study found elevated GM130 expression in lung adenocarcinoma patients, revealing its protective effect on patient survival (Li et al. 2022). These conflicting reports underscore the complexity of the relationship between GA fragmentation and cellular fate, emphasizing the need for a comprehensive understanding of the changes in GA morphology.

GA fragmentation is not only a feature observed in cancer but also in neurodegenerative diseases, including AD, amyotrophic lateral sclerosis (ALS), and Parkinson’s disease (PD) (Haase and Rabouille 2015). In neurons, the GA plays a crucial role in regulating anterograde trafficking to release neurotransmitters (Martinez-Menarguez et al. 2019). The fragmented morphology of the GA is predominantly observed in hyperexcitable neurons and neuronal hyperactivity, such as in ALS (Thayer et al. 2013; Weskamp et al. 2020). Unlike the irreversible GA fragmentation seen in apoptosis, GA fragmentation induced by neuronal hyperactivity causing diseases is reversible when neuronal activity returns to normal. In AD, CDK5 overactivation leads to the phosphorylated GM130 and GRASP65, resulting in a GA fragmentation (Liu et al. 2019). Interestingly, nicotine intake has been shown to protect against certain neuronal diseases, such as AD and PD (Piao et al. 2009), and can induce GA fragmentation through α4β2 receptors, which have a high affinity for nicotine. Remarkably, despite the fragmented GA morphology, cargo can still be transported through this GA fragmentation (Govind et al. 2021).

## The function of GA fragmentation stem cell activation and wound repair

The GA undergoes repositioning and fragmentation during key cellular processes, such as cell differentiation. Various stem cell types, including myoblast, urothelial stem cells, and neural stem cells, initially maintain a perinuclear GA cluster anchored by microtubules, which undergo significant shifts as they mature. For instance, myoblast reposition their GA from a perinuclear position to an even distributed configuration as they differentiate into myotubes, with the repositioning being actin-dependent and driven by Myosin VI (Lu et al. 2001; Percival and Froehner 2007). Similarly, neural stem cells initially have perinuclear GA. During development, radial glial cells (RG) undergo apical-basal repositioning in various contexts, including Drosophila quiescent neural stem cells reactivation (Gujar et al. 2023) and neurogenesis from induced pluripotent stem cell (iPSC) (Scharaw et al. 2023). These dynamic changes in GA positioning and morphology raise intriguing questions about their potential role in stem cell contributions to wound healing. For example, Scharaw et al. reported that iPSCs regulate the morphology and orientation of the GA to match the niche Paneth cells (Scharaw et al. 2023), suggesting a potential mechanism for targeted protein delivery within stem cell niches.

Moreover, the ability of stem cells to switch between quiescent and proliferative states is fundamental for tissue homeostasis. Adult neural stem cells (NSCs), primarily in a quiescent state, can be awakened by physiological cues such as injury or nutritional shifts (Urbán et al. 2019). Drosophila larval brain NSCs, also known as neuroblasts, serve as a powerful model to study the mechanisms governing quiescence and reactivation in vivo. These neuroblasts enter a quiescence state post-embryogenesis and resume proliferation after larval hatching, triggered by dietary amino acids. Their reactivation relies on an evolutionarily conserved insulin growth factor (IGF) signaling pathway and microtubule-rich extensions. Recent studies suggest that the GA and its outposts (GOP) can act as a microtubule-organizing center (MTOC) in quiescent NSCs (Valenzuela et al. 2020), with Golgi-resident GTPase ARF1 and its guanine nucleotide exchange factor (GEF) Sec71 playing a pivotal role in promoting NSC reactivation and regeneration (Gujar et al. 2023). These findings highlight the essential role of GA in regeneration after injury in vivo.

**Fig. 3:**
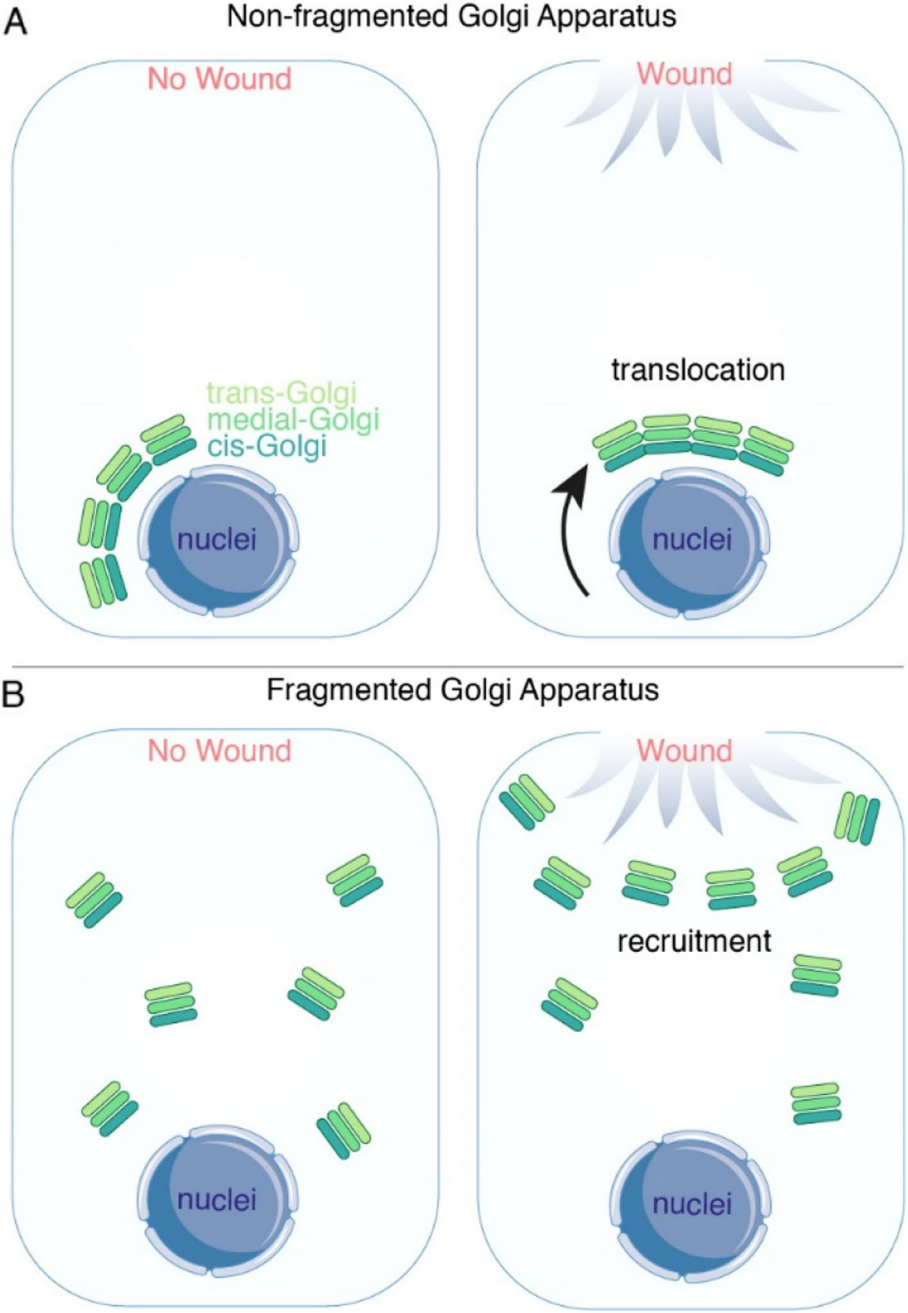
Illustration of GA morphology in response to tissue and plasma membrane damage. **A** In mammalian cells, the GA is localized on the perinuclear region (left). However, when a wound is induced to the tissue or cell culture, the GA repolarizes towards the direction of the wound, guiding cell migration toward the wound to facilitate wound closure (Right). **B** In various cell types, such as neurons, muscle cells, and tumor cells, as well as in some lower organisms, the GA is scattered throughout the cytosol. As an example, the GA of the adult *C. elegans* hyp7. In the case of hyp7, damage to the plasma membrane triggers the enrichment of GA at the wound site to promote PM repair

The GA also plays a crucial role in wound repair in vitro, potentially by ensuring the targeted delivery of essential proteins and lipids. Upon induction of a wound, the GA acts as a conductor of cell polarity, facilitating precise trafficking of key proteins, guiding cell migration, and orchestration the wound closure (Bisel et al. 2013; Darido and Jane 2013; Yadav et al. 2009) (Fig. 3A). Depletion of GA organization-related proteins such as Golgin160 or GMAP210 disrupts the GA perinuclear distribution, impacting polarized trafficking mechanisms crucial for directed protein delivery during wound healing. Importantly, it is observed that there is no enrichment of Vesicular Stomatitis Virus G protein (VSV-G) towards the wound in these cells after scratching the wound (Yadav et al. 2009). Unlike the microtubule-dependent GA fragmentation observed during cell division, GA remodeling in migrating cells is actin-dependent (Valente and Colanzi 2015), as observed in a scratch wounding assay in astrocytes, where CDC-42 activation at the leading edge promotes GA reorientation and polarization towards the wound (Fig. 3A). Thus, these results highlight the importance of GA fragmentation in cellular wound repair. Understanding how stem cells regulate GA morphology and orientation in response to injury signals could offer novel therapeutic strategies for enhancing tissue wound repair and regeneration.

## The function of GA fragmentation in plasma membrane repair

The plasma membrane is a critical barrier protecting the cell from external factors. Any form of physical, chemical, or biological damage threatens cell survival. Skeletal muscle injury during exercise, radiation-induced injury to the cutaneous epithelial cell, microbial-induced damage, ischemia, and other factors can compromise its integrity, jeopardizing cell survival (Ammendolia et al. 2021). In response to these threats, cells must activate the repair processes to ensure cell survival. Any defect in the PM repair is associated with diseases such as muscular dystrophy, cardiovascular diseases, and neuronal diseases.

When the plasma membrane is damaged, various mechanisms contribute to the repair process, including membrane tension, endocytosis, exocytosis, patch, and membrane shedding (Xu et al. 2023). The specific repair mechanisms employed depend on the cell type, wound type, and size (Cai et al. 2009; Jimenez et al. 2014). Following repair, membrane remodeling occurs, involving alterations in membrane components to restore their proximity to the undamaged PM (Ammendolia et al. 2021). Modifying and controlling PM repair signaling and mechanisms is crucial to maintain homeostasis and prevent pathological development. Notably, overactivation of the repair mechanism has been observed in cancer cells, showing a high ability to protect against the immune cell attack or during metastasis (Dias and Nylandsted 2021). However, whether the GA functions in repairing the damaged membrane remains largely unknown.

We use adult *C. elegans* hyp7 cell (large syncytium epidermal cell with 139 nuclei) as an in vivo model investigating the membrane wound response and repair (Chisholm and Hsiao 2012; Ma et al. 2021; Xu et al. 2022). Recently, we observed the GA is evenly distributed throughout the hyp7 cell (Table 1). Upon PM damage, the GA is recruited to the wound site, suggesting potential de-novo assembly at the wound site (Fig. 3B). This recruitment is supported by the evidence that inhibition of ER-GA anterograde transport blocks GA recruitment (Meng et al. 2023). Significantly, inhibition of GA recruitment to the wound site also hinders wound repair, leading to high animal lethality after membrane damage, highlighting the importance of GA in membrane repair in *C. elegans* epidermal cell (Meng et al. 2023).

Moreover, we identified ZC8.6, a GA-resident PI4Kα, and PPK-1, a PtdkIns4P5K, as crucial players in the PM repair process and highlighted their contribution to the generation of PtdIns4P to Phosphatidylinositol 4,5-bisphosphate (PtdIns4,5P_2_) at the wound site. GA recruitment facilitates polarized secretion toward the wounded PM, providing necessary membranes and PtdIns4P to restore the PtdIns4,5P_2_ level in the damaged PM (Meng et al. 2023). This finding is consistent with the requirement of RAB-6.2 for the transport of PtdIns4P to the damaged PM (Meng et al. 2023). Given the presence of similar GA structures in various cell types, such as skeletal muscle and cancer cells with fragmented GA, it is worth investigating whether GA translocation occurs in response to membrane damage and facilitates repair in muscle and cancer cells. Furthermore, understanding the translocation of GA may shed light on the mechanisms underlying the evasion of cell protective mechanisms observed in certain cancer cells during T cell attack and metastasis.

The biological significance of GA’s contribution to the PM PtdIns4,5P_2_ was overshadowed by the PM PtdIns4P pool in a study conducted using the tsA-201 cell line (Dickson et al. 2014). Yet in adult *C. elegans* hyp7, GA provides a PtdIns4P pool for PtdIns4,5P_2_ generation during PM damage. This could be due to the translocation capability of the adult *C. elegans* hyp7 non-perinuclear GA (Meng et al. 2023), yet not ruling out the different mechanisms to restore PtdIns4,5P_2_ under damaged PM condition. Therefore, investigating mechanisms in skeletal muscle injury and exploring the similarities in PM repair mechanisms among cells with similar GA morphologies present intriguing areas of study.

However, the role of GA in PM repair, particularly in skeletal muscle cells and carcinogenesis, has received limited attention. Skeletal muscle PM is frequently subjected to mechanical and chemical damage, which triggers repair mechanisms involving Annexins, dysferlin, SNAREs, and inflammatory signaling pathways (Allen et al. 2008; Demonbreun et al. 2016). Notably, Plasma membrane repair involves the requirement of a membrane source to restore the membrane integrity. Membrane fusion and patching are two of the proposed membrane repair machinery that require SNARE complex to complete (Andrews and Corrotte 2018). In *C. elegans*, the SYX-2/RIC-4/SEC-22 SNARE complex can promote membrane repair by facilitating membrane fusion (Shao et al. 2023). In the context of carcinogenesis, the GA morphology of cancer cells may show uncanonical distribution. Understanding the GA morphology in cancer cells and its impact on PM repair machinery remains an understudied aspect, holding potential insights into cancer cell resistance and evasion of cell death. The translocation of GA in this context could unveil unconventional perspectives on the organelle’s involvement in PM repair and polarized transport regulation during injury.

## Conclusions

Despite a general understanding of GA, its observation in diverse cell types, organisms, pathological conditions, or experimentally induced “fragmentation” reveals distinct patterns of GA distribution and morphology (Mogelsvang et al. 2003; Papanikou and Glick 2009; Petrosyan 2015; Petrosyan and Cheng 2013). This diversity raises the question of why different causes of GA fragmentation lead to varied GA morphologies. In contrast to the well-defined mechanism of mitochondrial fragmentation, which is conserved and serves a common biological function, the definition of GA fragmentation remains conflicted (Kleele et al. 2021).

Several key aspects must be addressed to establish a gold standard for defining GA fragmentation corresponding to a function. Firstly, the identification of inducers of fragmentation is essential. Secondly, a detailed examination of changes in GA morphology is required, including aspects such as ribbon unlinking, reduced stack number, cisternal fragmentation, or alterations in GA distribution. Lastly, understanding how the function of GA fragmentation, including changes in transport, cell polarity, protein modifications, and other cellular processes. Addressing these questions will enhance our understanding of GA fragmentation, its mechanisms, and its functional consequences, contributing to a more comprehensive understanding of the role of GA in cellular processes and disease progression.

On the other hand, the term “fragmentation” cannot be universally applied to all instances of GA morphological changes. Consider whether it involves ribbon unlinking, loss of stacked GA structure, or alteration in subcellular distribution for accurate GA morphology definition. Moreover, determining whether these changes in GA morphology promote cell survival, as observed in cancer progression, or lead to harm, as in apoptotic cells, is crucial. For example, the word “translocation” or “re-localization” could better describe the loss of GA perinuclear distribution (Khuntia et al. 2022), while “depolarization” may better describe the incapability of GA to face one direction for targeted transport (Jiang et al. 2017).

While changes in GA morphology are often linked with disease pathogenesis (cancer and neurodegenerative), some specific cell types, like skeletal muscle cells, yeast, and adult *C. elegans* hyp7 cells, naturally exhibit non-perinuclear or non-canonical GA morphologies. Understanding why these cell types retain such morphologies deserves further investigation. Additionally, the assessment of GA fragmentation must be improved based solely on morphology to determine a biological condition (Makhoul et al. 2019). Employing more sophisticated assays to evaluate the function of GA fragmentation is essential. Given that GA is involved in trafficking and protein modification, exploring whether GA morphology can serve as a parameter to determine the nature, fate, and diagnostic potential is intriguing. Clear classification of GA morphology changes can provide new insights and make breakthroughs in understanding the field’s unresolved physiological and pathological mechanisms.

## Data Availability

Not applicable.

## Abbreviations

AD: Alzheimer’s Disease
ALS: Amyotrophic Lateral Sclerosis
aRG: Apical radial glia
BFA: Brefeldin A
bRG: Basal radial glia
*C. elegans*: *Caenorhabditis elegans*
COPI: Coat protein complex I
COPII: Coat protein complex II
ER: Endoplasmic reticulum
GA: Golgi Apparatus
GAAP: Golgi apparatus anti-apoptotic proteins
GOP: Golgi Outpost
GRASP: Golgi reassembly stacking protein
MTOC: Microtubule organizing center
NMIIA: Non-muscle myosin IIA
NSF: N-ethylmaleimide sensitive fusion protein
PD: Parkinson’s Disease
PM: Plasma membrane
*P. pastoris*: *Pichia pastoris*
PtdIns4P: Phosphatidylinositol 4-phosphate
PtdkIns4P5K: Phosphatidylinositol 4-phosphate-5-kinase
PtdIns4,5P_2_: Phosphatidylinositol 4,5-bisphosphate
RNAi: RNA interference
*S. cerevisiae*: *Saccharomyces cerevisiae*
siRNA: Short interfering RNA
SNARE: Soluble N-ethylmalemide-sensitive-factor attachment protein receptor
tER: Transitional endoplasmic reticulum
VSV-G: Vesicular stomatitis virus G protein
